# Acute pancreatitis in patients with pancreatic cancer: Timing of surgery and survival duration

**DOI:** 10.1097/MD.0000000000005908

**Published:** 2017-01-20

**Authors:** Shaojun Li, Bole Tian

**Affiliations:** Department of Hepatobiliopancreatic Surgery, West China Hospital, Sichuan University, Chengdu, Sichuan Province, China.

**Keywords:** acute pancreatitis, complications, pancreatic cancer, survival rate, timing of surgery

## Abstract

Acute pancreatitis (AP) is a rare manifestation of pancreatic cancer (PC). The relationship between AP and PC remains less distinct.

From January 2009 to November 2015, 47consecutive patients with PC who presented with AP were reviewed for this study. Clinical features, clinicopathologic variables, postoperative complications, and follow-up evaluations of patients were documented in detail from our database. In order to identify cutoff threshold time for surgery, receiver operating curve (ROC) was built according to patients with or without postoperative complications. Cumulative rate of survival was calculated by using the Kaplan–Meier method. The study was conducted in accordance with the principles of the Declaration of Helsinki and the guidelines of West China Hospital.

This study included 35 men (74.5%) and 12 women (25.5%) (mean age: 52 years), with a median follow-up of 40 months. AP was clinically mild in 45 (95.7%) and severe in 2 (4.3%). The diagnosis of PC was delayed by 2 to 660 days (median 101 days). Thirty-nine (83.0%) cases underwent surgery. Eight (17.0%) cases performed biopsies only. Of 39 patients, radical surgery was performed in 32 (82.1%) cases and palliative in 7 (19.9%) cases. Two (8.0%) patients were needed for vascular resection and reconstruction. Postoperative complications occurred in 12 (30.8%) patients. About 24.5 days was the best cutoff point, with an area under curve (AUC) of 0.727 (*P* = 0.025, 95% confidence interval: 0.555–0.8999). The survival rate of patients at 1 year was 23.4%. The median survival in patients with vascular resection and reconstruction was 18 months, compared with 10 months in patients without vascular resection (*P* = 0.042). For the primary stage (T), Tix was identified in 3 patients, the survival of whom were 5, 28, 50 months, respectively. And 2 of them were still alive at the follow-up period.

The severity of AP was mainly mild. Surgical intervention after 24.5 days may benefit for reducing postoperative complications. Patients with vascular resection and reconstruction, thus achieving tumor-free margins, had a long-time survival.

## 1 Introduction

Pancreatic cancer (PC) is an aggressive disease and ranks fourth in cancer-related mortality in the United States.^[1]^ The prognosis of PC is dismal due to its asymptomatic nature in the early stages. Less than 30% of patients with PC were impossible to remove the tumors when classical clinical findings were present.^[2]^ One approach to cure PC is early diagnosis and radical surgery. Disappointingly, there is a lack of effective intervention for early diagnosis of PC at present. Once clinical presentations of PC present with weight loss, abdominal or back pain, and jaundice, it may be a sign of late stage.

Recently, it has been described that acute pancreatitis (AP) is an early symptom of PC. Mujica et al^[3]^ reported that 1-year overall survival rate was 28% in patients with PC presenting with AP and 20% in patients with PC. However, AP is a rare manifestation of PC. Most patients may be misdiagnosed as AP and delayed in cancer diagnosis. Little is known of the relationship between clinical features of AP and PC. The objectives of this report were to identify the clinical characteristics, the optimal timing of surgical intervention, and survival.

## 2 Methods

### 2.1 Patient selection

Forty-seven consecutive patients with PC who presented with AP attending our institution between January 2009 and November 2015 were reviewed for this study. There were 35 men (74.5%) and 12 women (25.5%), with a mean age of 51.7 ± 10.7 years (range 21–73 years). All patients on admission had presented with AP. Exclusion criteria were as follows: patients diagnosed with PC >2 years of AP diagnosis; patients diagnosed as AP in other medical centers; tumors histologically proved to be pancreatic neuroendocrine neoplasms (pNENs) and pancreatic cystic neoplasm (PCN).

### 2.2 Definitions

The diagnosis of AP is based on severe abdominal pain together with serum amylase and/or lipase 3 or more times the upper limit of normal and/or abdominal imaging demonstrating relevant morphological changes. All the selected patients with PC had to be proved by histological examination of surgical specimens or fine needle biopsy. Postoperative pancreatic fistula were graded A, B, C according to the International Study Group on Pancreatic Fistula (ISGPF).^[4]^ Delayed gastric emptying (DGE) was diagnosed according to the International Study Group of Pancreatic Surgery (ISGPS) criteria.^[5]^ Alcohol abuse was defined as patients with an intake of more than 80 g/day for more than 2 years. Early postoperative complications were defined as occurring within 30 days after operation. According to the timing of surgery identified by receiver operating characteristic (ROC) curve, the surgical patients (n = 39) were classified into 2 groups: early surgery group (Surgery ≤24.5 days, n = 25) and late surgery group (Surgery after 24.5 days, n = 14).

### 2.3 Data collection

This is a retrospective cohort study. Some important data were obtained from our outpatient. For patients who did not visit the outpatient, we would contact with their family members by phone or mail to ascertain whether the patient had died. The clinical data including preoperative, intraoperative, and postoperative parameters were collected. Preoperative variables including age, gender, severity of AP, serum CA19–9 values were recorded. Intraoperative data such as American Society of Anesthesiologists score, surgical time, blood loss were recorded from the anesthesia record. Postoperative parameters included complications and adjuvant therapy.

### 2.4 Statistics

The SPSS software (version 17.0 SPSS) was used to analyze the outcome data. Two-sided Fisher exact tests, Mann–Whitney *U* tests, ROC curve analysis, and Chi-square tests were used as appropriate. Survival curves were plotted using the Kaplan–Meier method and survival data were analyzed using the log-rank test. A value of *P* < 0.05 was considered significant.

## 3 Results

**Figure 1:**
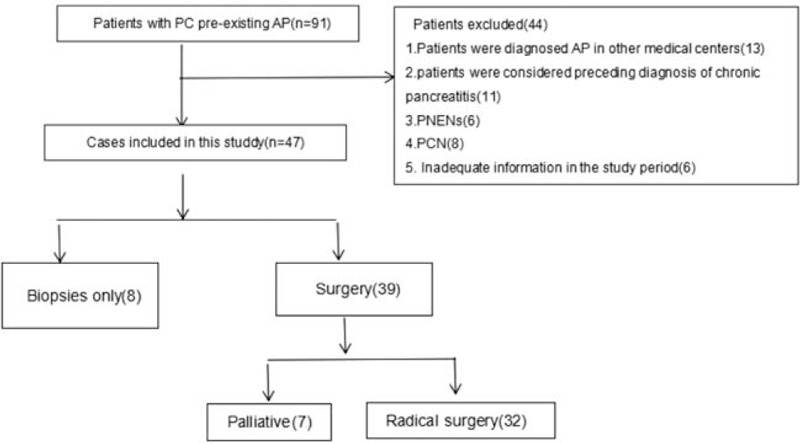
Different analyses of employed for different patients.

We retrospectively reviewed 91 patients with PC pre-existing AP. Of these, 13 patients were diagnosed with AP in other medical centers. Eleven patients were considered preceding diagnosis of chronic pancreatitis (CP). pNENs were found in 6 cases and PCN in 8 cases. Six cases had inadequate information in the study period. Of the 91 patients, 47 patients met the eligible criteria for study. Of these patients, radical surgery was performed in 32 (68.1%) cases, palliative surgery in 7 (14.9%) cases, and biopsies only in 8 (17.0%) cases (Fig. 1). The preoperative imaging included computed tomography (CT), magnetic resonance imaging (MRI)/magnetic resonance cholangiopancreatography (MRCP), endoscopic ultrasonography (EUS), and positron emission tomography/computer tomography (PET-CT). All the cases were evaluated by CT before surgery, 15 cases by MRI/MRCP, 4 cases by EUS, and 2 cases by PET-CT.

### 3.1 Demographic and disease-related data

**Figure 2:**
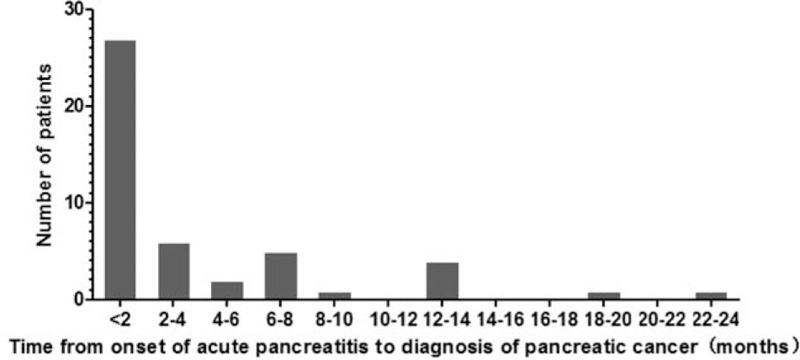
Duration of time between onset of acute pancreatitis (AP) and diagnosis of pancreatic cancer (PC). Twenty-seven were patients diagnosed in less than 2 months after acute pancreatitis diagnosis, and 6 patients diagnosed in more than 1 year.

The mean time interval between diagnosis and admission to the study was 108 ± 151 days (range 2–660 days). Twenty-seven (57.4%) patients were diagnosed in less than 2 months after AP diagnosis (Fig. 2). The main clinical characteristics of the study patients are summarized in Table 1. Abdominal pain occurred in 47 (100%) patients, jaundice in 4 (8.5%), and weight loss in 16 (34.0%). Forty-five (95.7%) patients with pancreatitis secondary to PC were clinically mild. But severe AP also occurred in 2 patients (4.3%). Forty-five (95.7%) patients had serum amylase and/or lipase 3 or more times the upper limit of normal, with mean level at 533.8 ± 603.0 (range 42–2852 IU/L) and 691.9 ± 547.3 (range 6.3–2853 IU/L), respectively. The lipase was within the normal range only in 2 patients. Before surgery, serum levels of CA19-9 were evaluated in all the selected patients. These patients had a mean CA 19-9 level of 242.3 ± 304.9U/mL (range 0.6–1000 U/mL). Tumor was mainly located in the head of pancreas (29 patients) and tumor ranged from 1.0 to 6.5 (mean 2.7) cm.

**Table 1:**
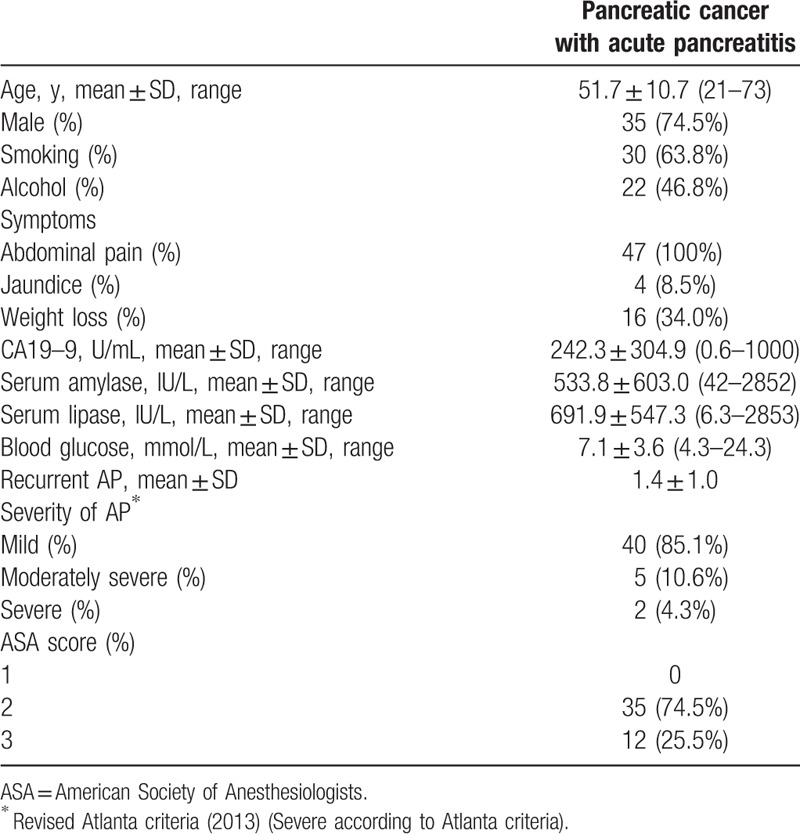
Clinical characteristics of the 47 patients with pancreatic cancer who present with acute pancreatitis as the first manifestation.

Diabetes was present in 6 patients (12.8%). Three required diet control, 1 required oral drugs, and 2 required insulin therapy. During follow-up, de novo diabetes developed in 4 (8.5%) additional patients who were controlled with oral antidiabetic agents.

### 3.2 Operative procedures and histopathologic evaluation

In total, 39 patients underwent surgery and 32 (68.1%) of 47 cases underwent radical surgery, including 22 standard pancreatoduodenectomy, 8 distal pancreatectomy, and 2 total pancreatectomy. Segmental resection of the superior mesenteric vein (SMV), portal vein (PV), or hepatic artery was performed in 14 of 39 patients.

For all the surgery patients, tumor stage was evaluated according to American Joint Committee on Cancer (AJCC) TNM Staging of Pancreatic Cancer (2010) guidelines (Table 2). Tix was identified in 3 patients, T1 in 2 patients, T2 in 11patients, T3 in 14 patients, and T4 in 9 patients. Regarding tumor grade, well or moderate was identified in 7 (17.9%) patients. Lymph node metastases were identified in 13 (33.3%) of the 39 patients. Table 3 summarizes the clinical characteristics of stage I patients (IA = 4; IB = 6) with PC presenting with AP.

**Table 2:**
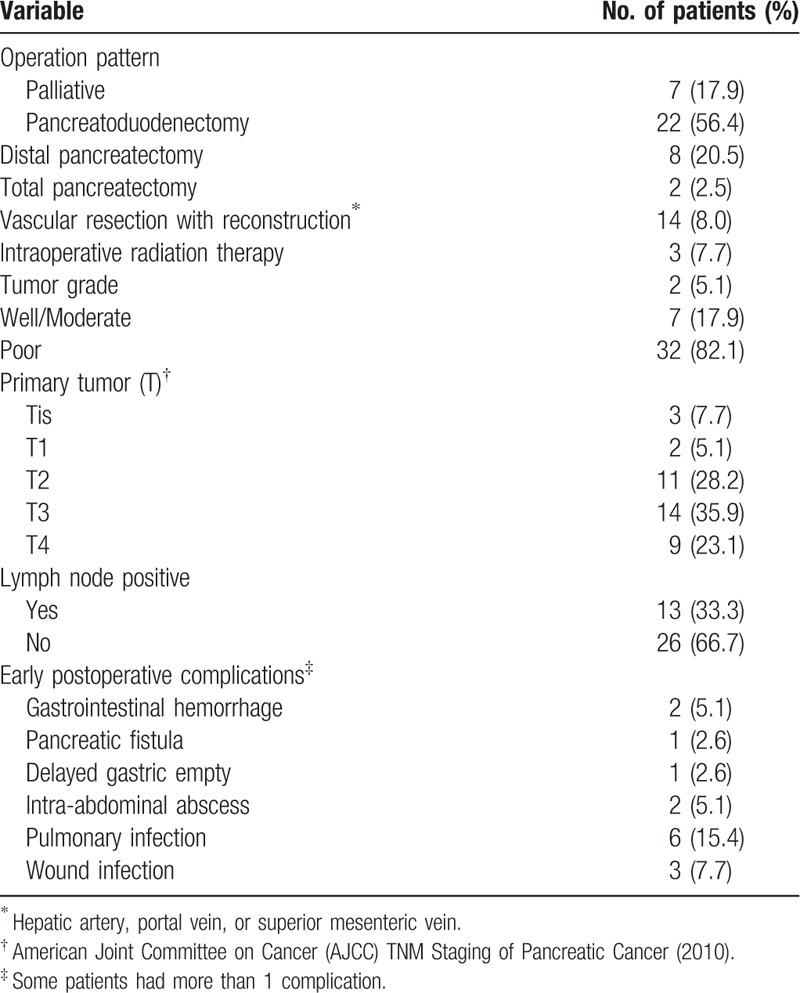
Operative procedures, tumor histology, and postoperative results of the 39 patients who received surgery.

**Table 3:**
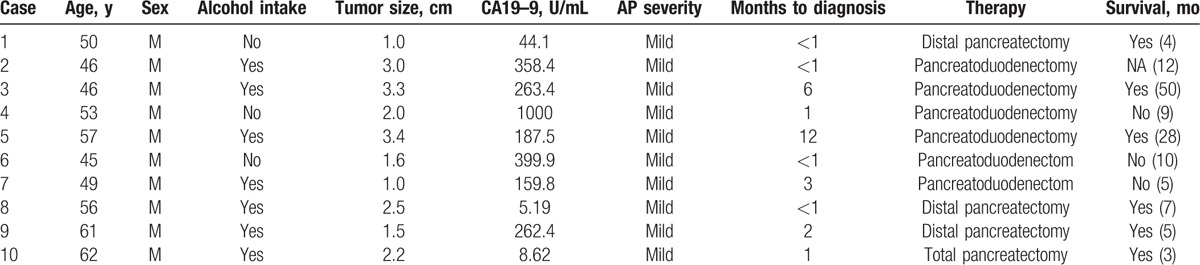
Clinical characteristics of stage I patients (IA = 4; IB = 6) with pancreatic cancer presenting with acute pancreatitis.

### 3.3 Timing of surgery and complications

**Figure 3:**
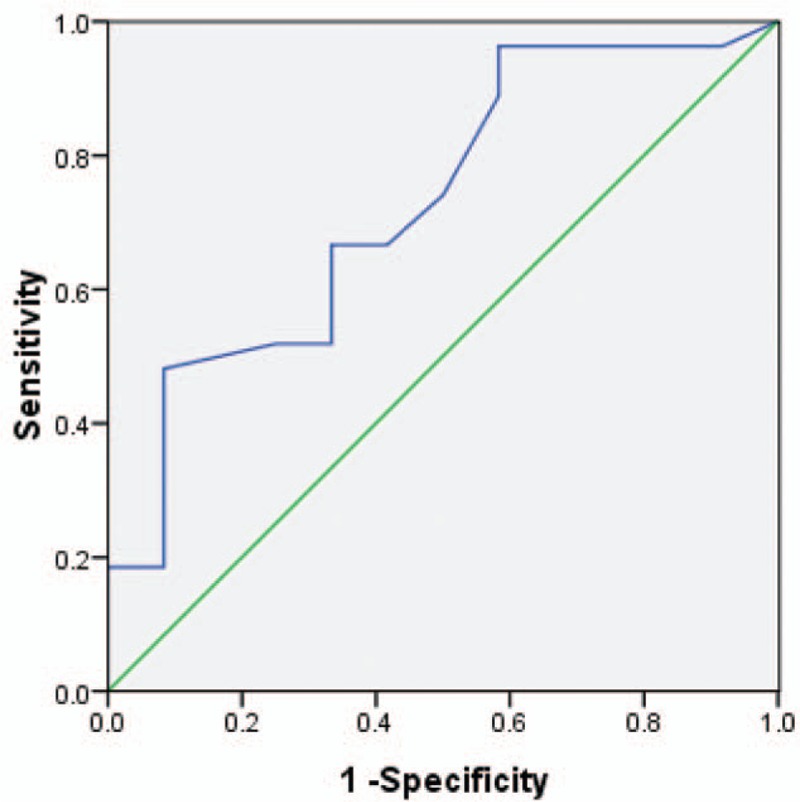
The best time cuttoff from acute pancreatitis attack to surgery (ROC curve: AUC = 0.727, *P* = 0.025, 95% CI: 0.555–0.899).

The time of surgery was calculated from the date of the first attack of AP (if pancreatitis was recurrent, then the time of surgery began with the latest attack.) to the surgery. ROC was used to analyze the best time cutoff from the first attack of AP to surgery according to early postoperative complications (Fig. 3). The best cutoff point of surgery was 24.5 days with an area under curve (AUC) of 0.727 [*P* = 0.025, 95% confidence interval (95% CI) 0.555–0.8999]. Twenty-five (64.1%) patients received surgery at or before 24.5 days from diagnosis of PC. Interestingly, early postoperative complications occurred in 12 patients. In the early surgery group (Surgery ≤24.5 days, n = 25), nearly half of the patients (44.0%) had complications, while in the late surgery group, only 7.1% patients had complications (*P* = 0.028) (Table 4). There was a significant postoperative intensive care unit stay in the 2 groups (*P* = 0.035).

**Table 4:**
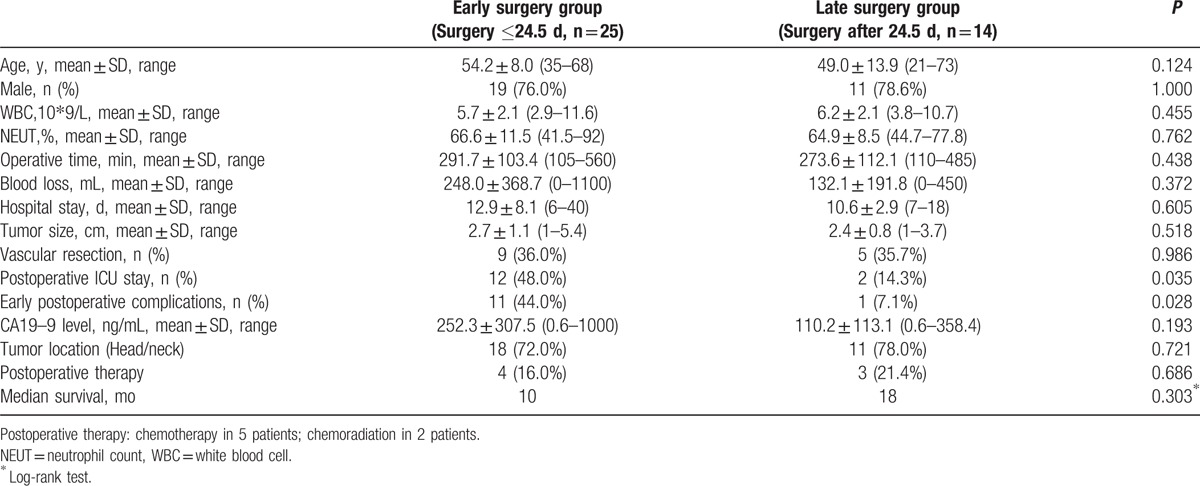
Compaction outcomes of selected patients who underwent surgery at 24.5 days or less with those who underwent later surgery.

### 3.4 Treatment and survival

**Figure 4:**
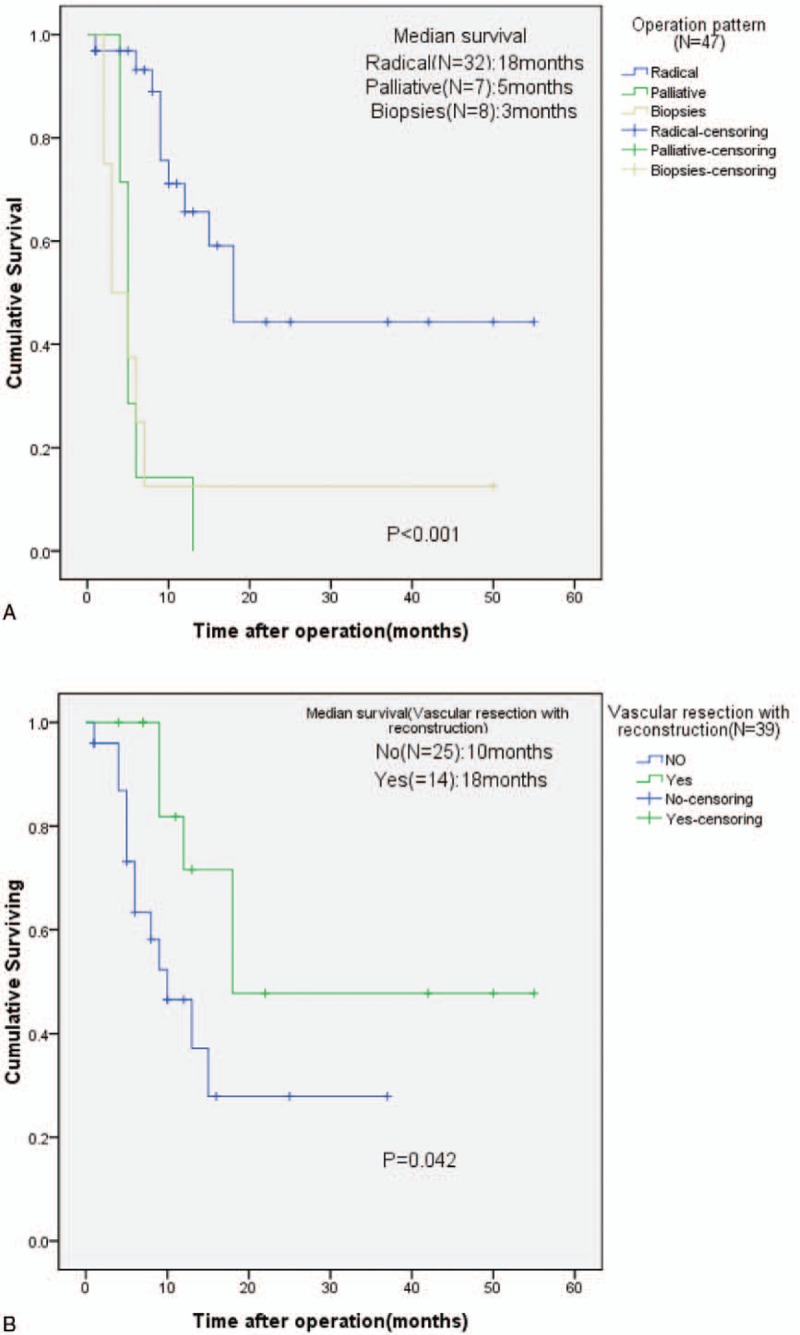
Kaplan–Meier survival curves in patients with pancreatic cancer who present with acute pancreatitis. (A) Survival of patients with different operation pattern (Log-rank test: *P* < 0.001). (B) Survival of patients who underwent surgery with or without vascular resection and reconstruction (Log-rank test: *P* = 0.042).

The median follow-up for patients was 24 months (range 4–54 months). The survival rate of patients with PC presenting initially with AP was 23.4% at 1 year. During postoperative therapy, 5 (10.6%) of the 47 patients received systemic chemotherapy and 2 (4.3%) received chemoradiation. Median survival was significantly different after resection compared with biopsies only (18 vs 3 months; *P* < 0.001) (Fig. 4A). Survival of patients with vascular resection did differ from survival of patients without vascular resection in our cohort (median survival 18 vs 10 months, respectively; *P* = 0.042) (Fig. 4B). Notably, there was no significant between 2 groups concerning postopative survival (Log-rank test, *P* = 0.303).

## 4 Discussion

This retrospective study supports the assumption that AP is the early presenting clinical symptom of PC. The rate of AP in PC patients varies widely and has been reported to range from 6.8% to 13.8%.^[6,7]^ Abdominal pain, weight loss, and jaundice were the main symptoms in patients with AP. We observed that abdominal pain occurred in 47 (100%) patients, jaundice in 4 (8.5%), and weight loss in 16 (34.0%). Eight (17.0%) of 47 patients before diagnosis of PC had more than 1 episode of AP, and the median number of episodes was 1.4, range 1 to 5. To our surprise, 85.1% of patients presenting with AP were mild, and only 2 of 47 patients severe, which was similar to the findings of study by Kohler and Lankisch.^[7]^

The underlying mechanisms and the nature course of this disease are unclear. Recently, one possible explanation was obstruction of pancreatic ducts,^[3,8]^ which resulted in the dilatation of main pancreatic duct and activated pancreatic enzymes. A similar observation was also made by other studies^[9,10]^ in patients with intraductal papillary mucinous neoplasms (IPMNs) presenting with AP. They believed that AP recurring after IPMN resection was the result of sudden obstruction of the main duct by abundant mucus secretion. Moreover, Kimura et al^[6]^ reported that PC may produce some chemical mediators, which may be responsible for AP. However, in our study, not all the cases present with dilatation of main pancreatic duct (6 patients). Why did AP happen in the absence of main pancreatic duct obstruction? Pelletier et al^[11]^ reported that the slow growth of PC may not narrow the main pancreatic duct; thus, AP did not occur frequently. Although the link between a history of AP and pancreatic tumors was uncertain in human body, animal studies have identified that AP could markedly accelerate PC development. Carrière et al^[12]^ made a mouse model and found that oncogenic Kras was activated in pancreatic acinar cell. All mouse subjected to two brief episodes of AP. They concluded that acute inflammation of the pancreas dramatically enhanced the risk for pancreatic malignant transformation.

Up to now, there is no guideline concerning the timing of surgical intervention in patients with PC pre-existing AP. AP is still a challenging disease with a high rate of morbidity and mortality. Most patients with mild course can be treated conservatively, and the incidence of severity occurred in almost 20%.^[13]^ AP has 2 clinical phase. The first phase is systemic inflammatory response syndrome (SIRS), which occurs 1 to 2 weeks after onset of the disease. The second phase occurs after 2 weeks, which is called counteractive anti-inflammatory response syndrome (CARS).^[14]^ Previous studies have demonstrated that it may benefit for patients who develop infection of necrosis to perform surgery in 4 weeks after onset of diagnosis of AP.^[15,16]^ In our study, 85.1% of patients were clinically mild. Patients with signs of mild AP were successfully treated by a conservative approach. Only 4.3% patients developed severe AP. Although patients with severe AP were admitted to an intensive care unit, no mortality happened. Infected necrosis was found in 2 patients and required necrosectomy at the same of distal pancreatectomy.

Although previous studies concerning optimal treatment strategy of patients with PC pre-existing AP have yet to be defined, our study suggested that 24.5 days was the best cutoff point of surgery, with an AUC of 0.727 (*P* = 0.025, 95% CI 0.555–0.8999). Over half of the patients (64.1%) received surgery at or before 24.5 days from diagnosis of PC. Surprisingly, the time of surgical intervention was significantly associated with postoperative complications (*P* = 0.028). Bhatia et al^[17]^ reported that in the nature course of AP, pancreatic acinar cells could produce and release inflammation and cytokines, such as TNF-α. They believed that the severity of the AP was mainly determined by the type of acinar cell death (apoptosis or necrosis) as well as by the systemic inflammatory response mediated. Patients might die from severe attack after surviving in the initial phase.^[18]^ The exaggerated secondary inflammatory response caused by minor event such as chest infection could contribute to multiple system organ failure, even death.^[19]^ In our series, we found that in the early surgery group, 44.0% of patients had complications. Regardless of the fact that some of these complications were not life-threatening events, the course of enhanced recovery after surgery (ERAS) was greatly influenced. All in all, early surgery may be not helpful to the recovery of patients.

With respect to the effect of surgery on survival in the patients with AP secondary to PC, 1-year survival rate was 23.4%, which was lower than previous study.^[3]^ No significant difference was found in tumor grade, intraoperative radiation therapy, tumor size, postoperation therapy, and lymph node positive. However, operation pattern, tumor stage, and vascular resection with reconstruction were found to be associated with overall survival. It was widely accepted that radical surgery was the only curative treatment option for PC. The median survival rates of patients who underwent resection was 20 to 24 months, compared with 3 to 6months in unresectable patients.^[20]^ There was a positive association between the overall survival rates and tumor stages (*P* = 0.001). Median survival for stage 0/I PC was higher than stage IV (10 vs 5 months).

The incidence of vascular resection varied widely, from 3% to 88%^[21–23]^ and the median survival after PV and/or SMV resection varied from 3 to 22 months.^[24–26]^ Furthermore, prior studies had found that the patients who needed for vascular resection did not improve the rate of survival.^[27,28]^ However, in our study, patients who had vascular resection with reconstruction had been associated with significantly better survival than those who had not (*P* = 0.042). And the median survival in resection patients was 18 months. Our conclusions could be explained as the follow reasons. Intraoperative radiation therapy and postoperative therapy including chemotherapy or chemoradiotherapy were available for patients with long-term survival. Kaneoka et al^[29]^ had investigated 81 patients with PC and 42 patients underwent PV/SMV resection. Although no significant difference in the survival rates between patients with vascular resection and patients who underwent standard PD only was observed, the length of PV/SMV resection < 3 cm seemed to had long-term survival. In our series, only 5 patients had the length of vascular resection more than 3 cm. So, it may be not surprising that the median survival was a litter longer than others. In addition, in our study, the aim concerning vascular resection and reconstruction was mainly to require R0 resection (in 14 patients). The fact that these patients with radical resection had received tumor-free resection margins, in some extent, suggests that patients having an earlier stage of cancer and AP might be the first clinical symptom.

As AP is a rare manifestation of PC, these groups of patients are usually misdiagnosed. In the present study, the causes of misdiagnosis are unclear. Generally, patients with PC was diagnosed in the first year after AP.^[30]^ In our study, the diagnosis of PC was delayed by 2 to 660 days (median 101 days). Minato et al^[31]^ suspected that diffuse pancreatic inflammation might have masked the presence of an underlying lesion in the pancreas or a small-sized tumor may preclude an early diagnosis of cancer. Another cause might be that a pancreatic mass was difficult to found on the images in the early stage of cancer. The patients diagnosed PC due to AP markedly accelerating PC development. Noticeably, in our study, four cases were preoperatively evaluated by EUS and 2 cases by PET-CT. The 6 patients had no tumor on images. Of the 6 patients, Tix was identified in 3 patients, the survival of whom were 5, 28, and 50 months, respectively. And, 2 of them were still alive at the follow-up period.

Our study has several limitations. First, the correlation between the topography of PC and the occurrence of AP is not clearly interpreted because of the retrospective nature of the study. Second, we could not include all patients in the analysis, as some patients diagnosed with PC >2 years may present with CP. Due to missing data on some important variables, patients diagnosed as AP in other medical center were excluded from the study. Furthermore, the sample size was small, and the time of our follow-up period was short. Hence, further studies are urgently needed.

In conclusion, AP could reveal PC at an earlier stage. Surgery after 24.5 days may benefit to patients. However, the time of surgery is mainly determined by the clinical condition of the patient. Patients with vascular resection and reconstruction had a long-time survival.
